# Preconditioning of murine mesenchymal stem cells synergistically enhanced immunomodulation and osteogenesis

**DOI:** 10.1186/s13287-017-0730-z

**Published:** 2017-12-06

**Authors:** Tzuhua Lin, Jukka Pajarinen, Akira Nabeshima, Laura Lu, Karthik Nathan, Eemeli Jämsen, Zhenyu Yao, Stuart B. Goodman

**Affiliations:** 1 Department of Orthopaedic Surgery, Stanford University School of Medicine, 450 Broadway Street, Redwood City, CA 94063 USA; 2 Bioengineering, Stanford University, Stanford, CA USA

**Keywords:** Mesenchymal stem cells, Immunomodulation, Macrophage polarization, Osteogenesis, Prostaglandin E2

## Abstract

**Background:**

Mesenchymal stem cells (MSCs) are capable of immunomodulation and tissue regeneration, highlighting their potential translational application for treating inflammatory bone disorders. MSC-mediated immunomodulation is regulated by proinflammatory cytokines and pathogen-associated molecular patterns such as lipopolysaccharide (LPS). Previous studies showed that MSCs exposed to interferon gamma (IFN-γ) and the proinflammatory cytokine tumor necrosis factor alpha (TNF-α) synergistically suppressed T-cell activation.

**Methods:**

In the current study, we developed a novel preconditioning strategy for MSCs using LPS plus TNF-α to optimize the immunomodulating ability of MSCs on macrophage polarization.

**Results:**

Preconditioned MSCs enhanced anti-inflammatory M2 macrophage marker expression (Arginase 1 and CD206) and decreased inflammatory M1 macrophage marker (TNF-α/IL-1Ra) expression using an in-vitro coculture model. Immunomodulation of MSCs on macrophages was significantly increased compared to the combination of IFN-γ plus TNF-α or single treatment controls. Increased osteogenic differentiation including alkaline phosphate activity and matrix mineralization was only observed in the LPS plus TNF-α preconditioned MSCs. Mechanistic studies showed that increased prostaglandin E2 (PGE2) production was associated with enhanced Arginase 1 expression. Selective cyclooxygenase-2 inhibition by Celecoxib decreased PGE2 production and Arginase 1 expression in cocultured macrophages.

**Conclusions:**

The novel preconditioned MSCs have increased immunomodulation and bone regeneration potential and could be applied to the treatment of inflammatory bone disorders including periprosthetic osteolysis, fracture healing/nonunions, and osteonecrosis.

**Electronic supplementary material:**

The online version of this article (doi:10.1186/s13287-017-0730-z) contains supplementary material, which is available to authorized users.

## Background

Mesenchymal stem cells (MSCs) have been specifically defined by the International Society for Cellular Therapy [1]. Potential translational applications of MSCs are highlighted by their properties for immunomodulation and tissue regeneration including those that relate to bone. MSC-based cell therapies have been applied to clinical trials of inflammatory disorders including graft-versus-host disease, diabetes, myocardial infarction, and osteoarthritis [2].

MSC-based therapies may have great potential for treatment of inflammatory bone disorders. Inflammation has a crucial but distinct role in bone healing and the remodeling process [3]. For example, acute inflammation is necessary for successful fracture healing and bone regeneration [4]. Depletion of macrophages greatly impaired bone healing in a murine bone injury model [5]. However, unresolved inflammation with excessive proinflammatory cytokines and chemokines mainly secreted by macrophages leads to impaired bone formation and increased bone destruction (osteolysis) [6]. In addition, sequential exposure of MSC-lineage cells to inflammatory M1 macrophages (tumor necrosis factor alpha (TNF-α)/interleukin-1 receptor antagonist (IL-1Ra)^high^) and anti-inflammatory M2 macrophages (Arginase 1 (Arg1)^high^ and CD206^high^) further enhanced osteogenic differentiation in vitro [7]. Therefore, stimulation of MSCs by inflammatory signals and sequential modulation of macrophage polarization status is crucial for optimizing bone formation. Studies using transgenic animals demonstrated that NF-κB signaling (a major downstream signaling of inflammatory stimulation) negatively regulated bone formation [8]; however, the effects of inflammatory ligand stimulation on osteogenic differentiation of MSCs depended on the dose, type, and timing of the stimulation [9]. Exposure of MSCs to a lower dose (0.1–5 ng/ml) of TNF-α inhibited, while a higher dose (50 ng/ml) increased, osteogenic differentiation in vitro [10, 11]. The induction of osteogenesis mediated by inflammatory stimulation, including TNF-α and lipopolysaccharide (LPS), was only observed with early treatment of MSCs [12–16].

Previous studies have shown that in addition to osteogenic potential, MSC-mediated immunomodulation could be regulated by proinflammatory cytokines [17, 18] or bacterial endotoxin [19]. MSCs exposed to LPS attenuated the response to sepsis by upregulating IL-10 secretion in macrophages by secretion of prostaglandin E2 (PGE2) [19]. MSCs exposed to the combination of interferon gamma (IFN-γ) and proinflammatory cytokines such as TNF-α, but not by either IFN-γ or TNF-α alone, reduced T-lymphocyte-mediated and macrophage-mediated immune responses [17, 18]. These results suggest that stimulation by multiple inflammatory factors is required to enhance MSC-mediated immunomodulation in a synergistic manner. However, the application of IFN-γ plus TNF-α preconditioned MSCs in bone disorders could be limited by the potential detrimental effect of IFN-γ plus TNF-α on MSC self-renewal and lineage differentiation [20].

In the current study, we show that preconditioning of MSCs with TNF-α and LPS enhances the immunomodulatory properties of MSCs on M1 to M2 macrophage polarization and simultaneously increases the osteogenic differentiation of MSCs. The strategic use of preconditioned MSCs could be applied to inflammatory bone disorders and bone tissue engineering, as well as chronic inflammatory disorders with excessive macrophage-mediated proinflammatory activation.

## Methods

### Isolation of murine mesenchymal stem cells and macrophages

The methods of isolating murine bone marrow-derived MSCs and macrophages have been described previously [21, 22]. Stanford’s Administrative Panel on Laboratory Animal Care (APLAC) approved this isolation protocol (APLAC 17566). In brief, bone marrow was collected from the femurs and tibias of C57BL/6 J male mice 8–10 weeks old. Institutional guidelines for the care and use of laboratory animals were observed in all aspects of this project. For MSC isolation, the cells were carefully suspended and passed through a 70-μm strainer, spun down, and resuspended in α-MEM supplied with 10% MSC certified fetal bovine serum (FBS; Invitrogen) and antibiotic antimycotic solution (100 units of penicillin, 100 μg of streptomycin, and 0.25 μg of Amphotericin B per ml; Hyclone, Thermo Scientific). The fresh medium was replaced on the next day to remove the unattached cells (passage 1). The immunophenotype of isolated MSCs (Sca1^+^/CD105^+^/CD44^+^/CD45^–^/CD34^–^/CD11b^–^; Additional file 1: Figure S1) was characterized by LSR II flow cytometer (BD Bioscience) at passage 4. For macrophage isolation, the bone marrow cells were washed three times with culture medium (RPMI 1640 medium supplemented with 10% heat-inactivated FBS, and the antibiotic/antimycotic solution), resuspended in the culture medium containing 30% of L929 cells conditioned medium and 10 ng/ml mouse macrophage colony stimulation factor (M-CSF), and replated in T-175 culture flasks at a concentration of 4 × 10^7^ cells per flask. Cells were allowed to expand for 5–7 days, with a medium change on the second day to remove nonadherent cells. Macrophages were confirmed by double-positive staining of cells (> 98.5%) using the surface markers F4/80 and CD11b for flow cytometric analysis after day 7.

### Preconditioning of mesenchymal stem cells and coculture with macrophages

**Fig. 1:**
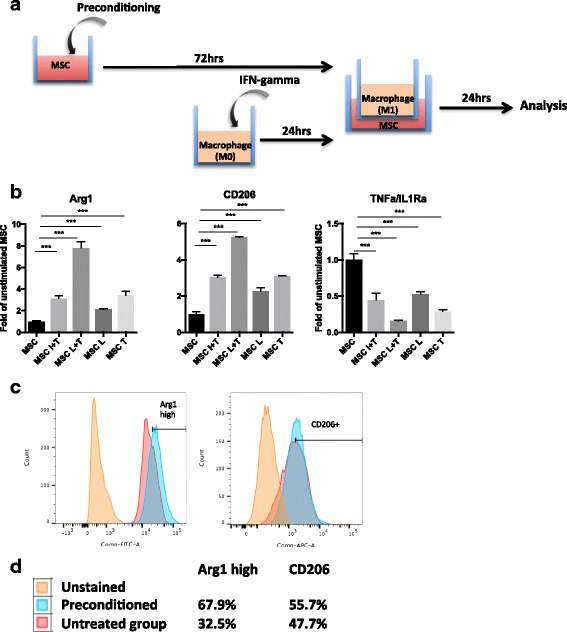
Immunomodulation of preconditioned MSCs on murine macrophages. **a** Preconditioning and coculture model. Murine MSCs were preconditioned with IFN-γ plus TNF-α (I + T, 20 ng/ml each), LPS (20 μg/ml) plus TNF-α (L + T), LPS alone (L), or TNF-α alone (T) for 3 days, and cocultured with M1 macrophages for 24 hours. **b** M2 (Arg1 and CD206) and M1 (TNF-α/IL-1Ra) macrophage marker expression in macrophages measured by quantitative PCR. Data presented as fold-change compared to macrophages cocultured with unstimulated control MSCs. **c**, **d** Expression of M2 macrophage markers (Arg1 and CD206) at protein level examined by flow cytometry. ***p < 0.005. Arg1 Arginase 1, IFN interferon, IL1Ra interleukin-1 receptor antagonist, MSC mesenchymal stem cell, TNF tumor necrosis factor

A summary of the experimental strategy is illustrated in Fig. 1a. At day 0, MSCs (1 × 10^4^ cells) were seeded in 24-well transwell plates (0.3 μm; Corning) in the bottom chamber with MSC growth medium. After cell attachment (day 1), the cells were treated with 20 ng/ml TNF-α and 1–20 μg/ml LPS for 3 days as indicated in the figure legend. The cells treated with 20 ng/ml TNF-α or 20 μg/ml LPS alone or the combination of 20 ng/ml IFN-γ and 20 ng/ml TNF-α [17] were used as controls. The dose of TNF-α and IFN-γ was determined according to previous studies of immunomodulation by MSCs [17, 18]. The dose of LPS was titrated (Additional file 1: Figure S1) in the range of previous studies on LPS-treated MSCs [10, 11]. Primary macrophages (2 × 10^3^ cells) were seeded to the insert of a separate transwell plate and polarized into the M1 phenotype by exposure to 20 ng/ml IFN-γ (day 3) for 24 hours. At day 4, MSCs and macrophages were washed three times with PBS and the inserts with the M1 macrophages were moved to the plates containing the preconditioned MSCs at the bottom of the well. This coculture was carried out in MSC growth medium for 24 hours. Celecoxib (25 μM) was used to block the cyclooxygenase (COX) signaling in selected groups for further mechanistic experiments. The medium was supplemented with 30 μg/ml polymyxin B1 to inactivate any trace amounts of LPS which could potentially impact the culture system.

### Quantitative PCR

Cellular RNA from macrophages in the coculture system was extracted using the RNeasy RNA purification kit (Qiagen, Valencia, CA, USA). RNA was reverse transcribed into complementary DNA (cDNA) using a high-capacity cDNA archive kit (Applied Biosystems, Foster City, CA, USA). Probes for 18 s rRNA, TNF-α, IL-1Ra, Arg1, and CD206 were purchased from Applied Biosystems. Reverse-transcriptase polymerase chain reaction (RT-PCR) was performed in an ABI 7900HT Sequencing Detection System (Applied Biosystems), using 18 s rRNA as the internal control. The –ΔΔCt relative quantization method was used to evaluate the gene expression level.

### Flow cytometry analysis of macrophage polarization markers

MSCs were seeded in 10-cm cell culture dishes and preconditioned or left untreated as described earlier. The conditioned media were collected 24 hours after the cells were washed and replaced with fresh medium. The conditioned media were diluted with macrophage growth medium at 1:1 ratio and used to treat IFN-γ-polarized macrophages for 24 hours. The cells were harvested into single cell suspension and stained with anti-CD206 antibody conjugated with PE (eBioscience), and processed by flow cytometry fixation and permeabilization buffer kit (R&D) for the following intracellular staining of Arg1 (FITC). Stained macrophages were analyzed by LSRII.

### Prostaglandin E2 quantification

Cellular supernatant from the coculture system was collected at the indicated time points. Concentration of PGE2 in the supernatant was measured by the PGE2 parameter assay kit (R&D system). The manufacturer’s protocol was followed carefully.

### Osteogenesis assay

After preconditioning (see Preconditioning of mesenchymal stem cells and coculture with macrophages for details), the MSCs were switched into osteogenic medium (α-MEM supplemented with 10% FBS, 100 nM dexamethasone, 10 mM β-glycerol phosphate, and 50 μM ascorbate-2-phosphate) supplemented with 30 μg/ml polymyxin B1 to inactivate the trace LPS in the system. Cell lysate at week 2 was used for the alkaline phosphatase (ALP) activity assay (QuantiChrome™ Alkaline phosphatase assay kit, Cat. No. DALP-250; Bioassay Systems, Hayward, CA, USA). Extracellular matrix mineralization by the murine MSCs was identified using Alizarin Red stain at week 3. Celecoxib (25 μM) was used to block the COX2 signaling at indicated time points described in figure legends. The results were photographed, and the staining was eluted by 10% cetylpyridinium chloride and quantified by measuring the absorbance at 562 nm.

### Statistical analysis

Nonpaired *t* tests were performed for data with two groups, and one-way ANOVA with Tukey’s post-hoc test was performed for data with three or more groups. The statistical analysis was conducted using Prism 7 (GraphPad Software, San Diego, CA, USA). Data are reported as mean ± standard error of the mean. The osteogenesis assay was performed with four replicates. Quantitative PCR was performed in triplicate. The experiments have been repeated twice in three or four replicates independently. *p* < 0.05 was chosen as the threshold of statistical significance.

## Results

### Preconditioned MSCs modulated macrophages into an anti-inflammatory phenotype

Murine MSCs were preconditioned and cocultured with murine bone marrow-derived macrophages as described in Methods and Fig. 1a. MSCs preconditioned with IFN-γ plus TNF-α were included as a positive control, because this conditioning has been demonstrated previously to enhance the modulation of both innate and adaptive immune responses [17, 18]. Activation of proinflammatory cytokines (TNF-α) also induces the production of anti-inflammatory cytokines (IL-1Ra) as a negative feedback mechanism to avoid overwhelming inflammation. The status of macrophage polarization was thus shown in the ratio of TNF-α/IL-1Ra expression [7, 23]. First, LPS dosage was titrated by treating MSCs with varying dosages of LPS (0.1–20 μg/ml) together with 20 ng/ml TNF-α [17, 18]. We found that preconditioning with 20 μg/ml LPS optimally enhanced the ability of MSCs to induce anti-inflammatory phenotypes in macrophages (Arg1^high^, CD206^high^, TNF-α/IL-1Ra^low^; Additional file 1: Figure S2). Preconditioned MSCs with LPS plus TNF-α synergistically enhanced M2 macrophage marker (Arg1^high^ and CD206^high^) and decreased M1 macrophage marker (TNF-α/IL-1Ra^low^) expression in the cocultured M1 macrophages (Fig. 1b). MSCs preconditioned with LPS (20 μg/ml) or TNF-α (20 ng/ml) alone, or with the combination of IFN-γ and TNF-α (20 ng/ml each), induced the anti-inflammatory phenotypes in M1 macrophages at lower levels. M2 marker expression in macrophages (Arg1 and CD206) at the protein level was further confirmed using the conditioned medium from MSCs (Fig. 1c, d). The Arg1^high^ population was increased from 32.5 to 67.9%, and the CD206^+^ population was increased from 47.7 to 55.7%, respectively. Together, the combination of LPS and TNF-α preconditioning further enhanced the immunomodulation by MSCs compared to other conditions.

### Preconditioned MSCs with LPS plus TNF-α enhanced osteogenic differentiation

**Fig. 2:**
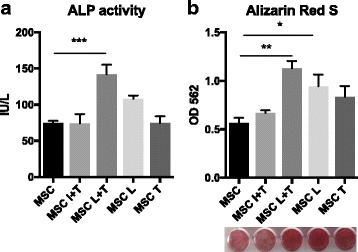
Increased osteogenic differentiation of MSCs preconditioned with LPS plus TNF-α. MSCs were preconditioned with IFN-γ plus TNF-α (I + T, 20 ng/ml each), LPS (20 μg/ml) plus TNF-α (L + T), LPS alone (L), or TNF-α alone (T) for 3 days in growth media, and replaced by osteogenic media for 3 weeks. The osteogenic differentiation ability was examined by ALP activity at week 2 (**a**) and bone mineralization (Alizarin Red staining) at week 3 (**b**). *p < 0.05, **p < 0.01, ***p < 0.005. ALP alkaline phosphatase, MSC mesenchymal stem cell

The osteogenic ability of MSCs using various preconditioning protocols was then examined. MSCs preconditioned with LPS plus TNF-α significantly enhanced ALP activity (*p* < 0.005) and bone mineralization (*p* < 0.01); however, MSCs preconditioned with LPS increased bone mineralization (*p* < 0.05) but had no significant effect on ALP activity (Fig. 2a, b). MSCs preconditioned with TNF-α alone or with IFN-γ plus TNF-α did not affect the ALP activity and mineralization.

### Increased prostaglandin E2 production in preconditioned MSCs is associated with enhanced Arginase 1 expression in macrophages

**Fig. 3:**
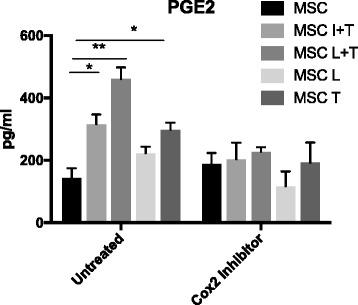
Induction of PGE2 secretion in preconditioned MSCs through the COX-2-dependent pathway. MSCs were preconditioned with IFN-γ plus TNF-α (I + T, 20 ng/ml each), LPS (20 μg/ml) plus TNF-α (L + T), LPS alone (L), or TNF-α alone (T) for 3 days, and replaced with fresh media with or without Celecoxib (25 μM) to modulate COX2 signaling. After 24 hours of incubation, the supernatants were collected, and PGE2 production was quantified by ELISA. *p < 0.05, **p < 0.01. COX2 cyclooxygenase-2, MSC mesenchymal stem cell, PGE2 prostaglandin E2

**Fig. 4:**
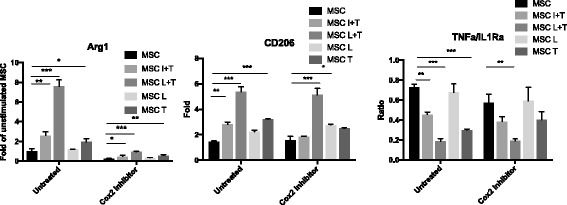
Immunomodulation ability of preconditioned MSCs mediated by PGE2 secretion. MSC/macrophage cocultured experiments were performed as described in Fig. 1a. Media were supplied with or without 25 μM Celecoxib to modulate COX2 signaling. M2 (Arg1 and CD206) and M1 (TNF-α/IL-1Ra) macrophage marker expression in macrophages was measured by quantitative PCR. Data presented as fold-change compared to macrophages cocultured with unstimulated control MSCs. *p < 0.05, **p < 0.01, ***p < 0.005. Arg1 Arginase 1, IL1Ra interleukin-1 receptor antagonist, MSC mesenchymal stem cell, TNF tumor necrosis factor

Inflammatory stimulation by TNF-α induces PGE2 production through the NF-κB/COX2-dependent pathway [24]. PGE2 secretion by MSCs is associated with the inhibition of inflammatory response [19, 25, 26] and the induction of osteogenic differentiation [27, 28]. We found that PGE2 production was significantly increased in the preconditioned MSCs including the groups treated with IFN-γ plus TNF-α, LPS plus TNF-α, and TNF-α alone, which could be reversed by a selective COX2 inhibitor (25 μM Celecoxib) (Fig. 3). MSCs preconditioned with LPS did not increase PGE2 production (*p* = 0.10). In the coculture model, inhibition of COX2 signaling by Celecoxib clearly suppressed Arg1 expression in macrophages cocultured with preconditioned MSCs (Fig. 4). However, the preconditioned MSC-mediated changes in CD206 and TNF-α/IL-1Ra were not affected by inhibition of COX2 signaling.

### Blocking the COX2/PGE2 pathway does not interfere with enhanced osteogenesis in preconditioned MSCs

**Fig. 5:**
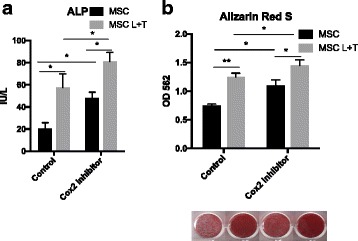
Inhibition of COX-2/PGE2 pathway increased osteogenesis in untreated or preconditioned MSCs. MSCs were preconditioned with LPS plus TNF-α in growth media, and replaced with osteogenic media for 3 weeks. Media were supplied with or without 25 μM Celecoxib during the first week of osteogenesis. Osteogenic differentiation ability examined by ALP activity at week 2 (**a**) and bone mineralization (Alizarin Red staining) at week 3 (**b**). *p < 0.05, **p < 0.01. ALP alkaline phosphatase, COX2 cyclooxygenase-2, MSC mesenchymal stem cell

We further examined the effects of increased PGE2 production on the osteogenic differentiation of preconditioned MSCs. Inhibition of the COX2/PGE2 pathway by Celecoxib during the first week of osteogenesis significantly enhanced ALP activity and mineralization in both the untreated and preconditioned (LPS plus TNF-α) MSCs (Fig. 5a, b). To further clarify how the COX2/PGE2 pathway regulated the osteogenic differentiation of MSCs in vitro, the MSCs were treated with Celecoxib at week 1, week 2, week 3, or continuously during the osteogenesis (weeks 1–3). Temporal inhibition of COX2 signaling enhanced bone mineralization in MSCs regardless of the treatment time points, while continuous treatment showed no significant effects compared to the untreated controls (Additional file 1: Figure S3).

## Discussion

Immunomodulation and tissue regeneration are fundamental mechanisms in MSC-based cellular therapy. These biological features are closely regulated in response to local environmental cues including the oxygen level, presence of growth factors and inflammatory cytokines, and other factors. Changes in the microenvironment during disease progression could provoke protective “pre-homeostatic” mechanisms in MSCs and further enhance their therapeutic efficiency [29–32]. Indeed, our current findings show that MSCs preconditioned with LPS plus TNF-α, macrophage-associated inflammatory mediators, synergistically enhanced M2 macrophage polarization and osteogenic differentiation.

Previous studies of MSC preconditioning have mainly focused on studying the effects of a single inflammatory mediator on MSCs [19, 29–33]. These approaches have clarified valuable information regarding the biological features of MSCs. However, as the complex tissue microenvironment contains multiple inflammatory factors, we speculated that it might be possible to further enhance the beneficial osteogenic and immunomodulatory properties of MSCs by exposing the cells to multiple inflammatory mediators. IFN-γ is mainly produced by activated Th1 lymphocytes and is a crucial regulator of both innate and adaptive immune response [34]. Ren et al. [17] demonstrated that MSC-mediated suppression of T-lymphocyte activation required the presence of IFN-γ and one of the proinflammatory cytokines TNF-α, IL-1α, or IL-1β. LPS is a potent ligand for macrophages that activates the proinflammatory response including TNF-α production via TLR4 signaling. TNF-α is a strong proinflammatory cytokine, produced in high amounts during inflammatory and immune reaction by various cells including M1 macrophages and Th1 cells. Our preconditioning strategy using high-dose LPS and TNF-α could incite physiological protective mechanisms against an overt inflammatory reaction and enhance the immunomodulatory properties of MSCs [19]. However, the proinflammatory stimulation-induced immunomodulation could also manipulate the infiltrated MSCs into tumor-derived MSCs and enhance tumor progression [35]. Indeed, as both TLR ligands and macrophage-derived TNF-α are likely to be present at the site of injury, they might work synergistically to induce the physiological transition from acute inflammation to tissue regeneration. Previous studies have shown that the application of MSCs preconditioned by a single factor improved tissue repair using in-vivo models [32, 36], demonstrating that ex-vivo conditioning with factors such as LPS/TNF-α can stably increase their therapeutic efficiency. The underlying mechanisms of synergistic effect by LPS plus TNF-α or other proinflammatory cytokines remain unclear.

Crosstalk between MSCs and macrophages including paracrine regulation and direct cell contact is crucial for effective immunomodulation and tissue regeneration [7, 19, 33]. Our current transwell coculture and conditioned medium treatment models demonstrated that paracrine factors secreted by the preconditioned MSCs were effective in modulating macrophage polarization from the inflammatory M1 phenotype toward the anti-inflammatory and tissue-regenerative M2 phenotype. Previous studies have shown that direct PGE2 treatment of macrophages increased M2 markers including Arg1, IL-10, and MMP9, and that this effect was blocked by PGE2 inhibitors [37–39]. In the current study, inhibition of the COX2/PGE2 pathway blocked the upregulation of M2-related Arg1 expression in macrophages, which is correlated with tissue repair. However, blocking of the COX2/PGE2 pathway had limited effects on CD206 expression and TNF-α/IL-1Ra ratio, suggesting that other secreted factors are also involved in immunomodulation. A previous proteomic analysis has identified 118 TNF-induced paracrine factors in human adipose-derived MSCs including various cytokines, chemokines, proteinases, and immune modulators such as long pentraxin 3 [40]. Analysis of paracrine factors secreted by the preconditioned MSCs is required to further clarify the underlying mechanisms of immunomodulation. Nemeth et al. [19] have shown that MSCs induced anti-inflammatory cytokine production in macrophages in the coculture system of direct contact or transwell (paracrine regulation only) models. The induction is higher with direct cell-to-cell contact compared to paracrine regulation alone, indicating that both physical contact and paracrine factors are involved in the MSC-mediated immunomodulation. Our study focused on the transwell system to clarify the MSC-mediated paracrine factors including nitric oxide and PGE2. Currently, the molecular candidates involved in the immunomodulation via physical contact between MSCs and macrophages remain unclear. Our data showed that MSC preconditioning alone increased osteogenic differentiation in the absence of macrophages in vitro. Sequential modulation of macrophage polarization status by the preconditioned MSCs could further enhance the osteogenic potential during this crosstalk [7].

MSCs can modulate adaptive and innate immunity by distinct mechanisms. Ren et al. [17] showed that MSC-mediated suppression of T-cell proliferation required iNOS-dependent nitric oxide production. Exposure of LPS plus TNF-α synergistically induced iNOS expression in MSCs (Additional file 1: Figure S4); however, selective inhibition of iNOS activity showed a limited effect on M2 macrophage polarization (data not shown). In addition, mechanistic studies demonstrated that the IFN-γ and TNF-α-induced immunomodulation in human MSCs is mediated by indoleamine 2,3-dioxygenase (IDO) instead of the iNOS pathway [18]. The different potential mechanisms to modulate macrophage polarization between human and mouse MSCs exposed to LPS plus TNF-α remain to be clarified.

The critical roles of COX2 in bone formation and repair have been demonstrated in COX2 knockout transgenic mouse [28, 41, 42], and by rat and rabbit models using nonselective nonsteroidal anti-inflammatory drugs (NSAIDs) [43–45]. However, clinical findings regarding the effects of COX-2 inhibitors and other NSAIDs on bone formation remain controversial [46–48]. Our data showed that transient inhibition of COX2 in MSCs by the selective COX-2 inhibitor Celecoxib increased osteogenesis, regardless of the treatment time points (Additional file 1: Figure S3). Interestingly, continuous treatment with Celecoxib had no significant effects on osteogenic differentiation. Other in-vitro studies showed that the suppressive effect of Celecoxib on osteogenesis was only observed in the presence of inflammatory environments, such as proinflammatory cytokines or macrophages [49, 50]. Therefore, the increased osteogenesis in MSCs treated with Celecoxib could be limited to our *in-vitro* model in the absence of other proinflammatory environmental factors.

Preconditioned MSCs could display proinflammatory or anti-inflammatory phenotypes in response to different ligands [51]. Transient exposure (< 1 hour) of MSCs to 10 ng/ml LPS led to proinflammatory “MSC1” phenotypes and increased T-cell activation in the coculture experiment. Comparatively, the “MSC2” phenotype exposed to 1 μg/ml poly I:C was able to suppress T-cell activation, enhance IDO expression, and increase PGE2 production [51]. Our data and other studies show that MSCs exposed to LPS (1–20 μg/ml) induced a protective anti-inflammatory effect on macrophages [19, 52]. The difference in the dose, exposure time, and affected cell types (T lymphocytes or macrophages) could determine the biological roles of LPS-preconditioned MSC.

## Conclusions

Our novel preconditioning strategy enhanced both immunomodulation and bone regeneration abilities of MSCs. This approach has great potential in the application of MSC-based therapy for the treatment of inflammatory bone disorders including periprosthetic osteolysis, fracture healing/nonunions, and osteonecrosis.

## Additional file

Additional file 1: Figure S1. Showing immunophenotypes of murine bone marrow-derived MSCs. Surface marker expression in murine MSCs at passage 4 analyzed by flow cytometry. **Figure S2.** showing titration of LPS dose for MSC preconditioning with TNF-α to modulate murine macrophage polarization. Murine MSCs were preconditioned with TNF-α (20 ng/ml) plus LPS (1–20 μg/ml) for 3 days, and cocultured with M1 macrophages for 24 hours. M2 (Arg1 and CD206) and M1 (TNF-α/IL-1Ra) macrophage marker expression in macrophages measured by quantitative PCR. Data presented as fold-change compared to macrophages cocultured with unstimulated control MSCs. **p* < 0.05, ***p* < 0.01, ****p* < 0.005. **Figure S3.** showing increased osteogenic differentiation in the MSCs with temporal inhibition of COX2 signaling. MSCs were treated with 25 μM Celecoxib in osteogenic media at indicated time points. Osteogenic differentiation ability examined by bone mineralization (Alizarin Red staining) at week 3. **p* < 0.05, ****p* < 0.005. **Figure S4.** showing increased iNOS expression in MSCs preconditioned with TNF-α and/or LPS. Murine MSCs were preconditioned with TNF-α (20 ng/ml) plus LPS (1–20 μg/ml) for 3 days, and washed out for 24 hours. Expression of iNOS determined by quantitative PCR. Data presented as fold-change compared to macrophages cocultured with unstimulated control MSCs. ****p* < 0.005. (PDF 3973 kb)

## Abbreviations

ALP: Alkaline phosphatase
ANOVA: Analysis of variance
APLAC: Administrative Panel on Laboratory Animal Care
Arg1: Arginase 1
COX2: Cyclooxygenase-2
FBS: Fetal bovine serum
FITC: Fluorescein isothiocyanate
IFN-γ: Interferon gamma
IL-10: Interleukin-10
IL-1Ra: Interleukin-1 receptor antagonist
LPS: Lipopolysaccharide
M-CSF: Macrophage colony stimulation factor
MSC: Mesenchymal stem cell
NF-κB: Nuclear factor-κB
PE: Phycoerythrin
PGE2: Prostaglandin E2
RPMI: Roswell Park Memorial Institute
RT-PCR: Reverse transcriptase-polymerase chain reaction
TNF-α: Tumor necrosis factor alpha
α-MEM: Alpha minimal essential medium
