# Supplementary figures and images for: Preconditioning of murine mesenchymal stem cells synergistically enhanced immunomodulation and osteogenesis
Source: Stem Cell Res Ther. 2017 Dec 6;8:277. doi: 10.1186/s13287-017-0730-z (PMC5719931; doi:10.1186/s13287-017-0730-z)

## Additional file 1: Figure S1

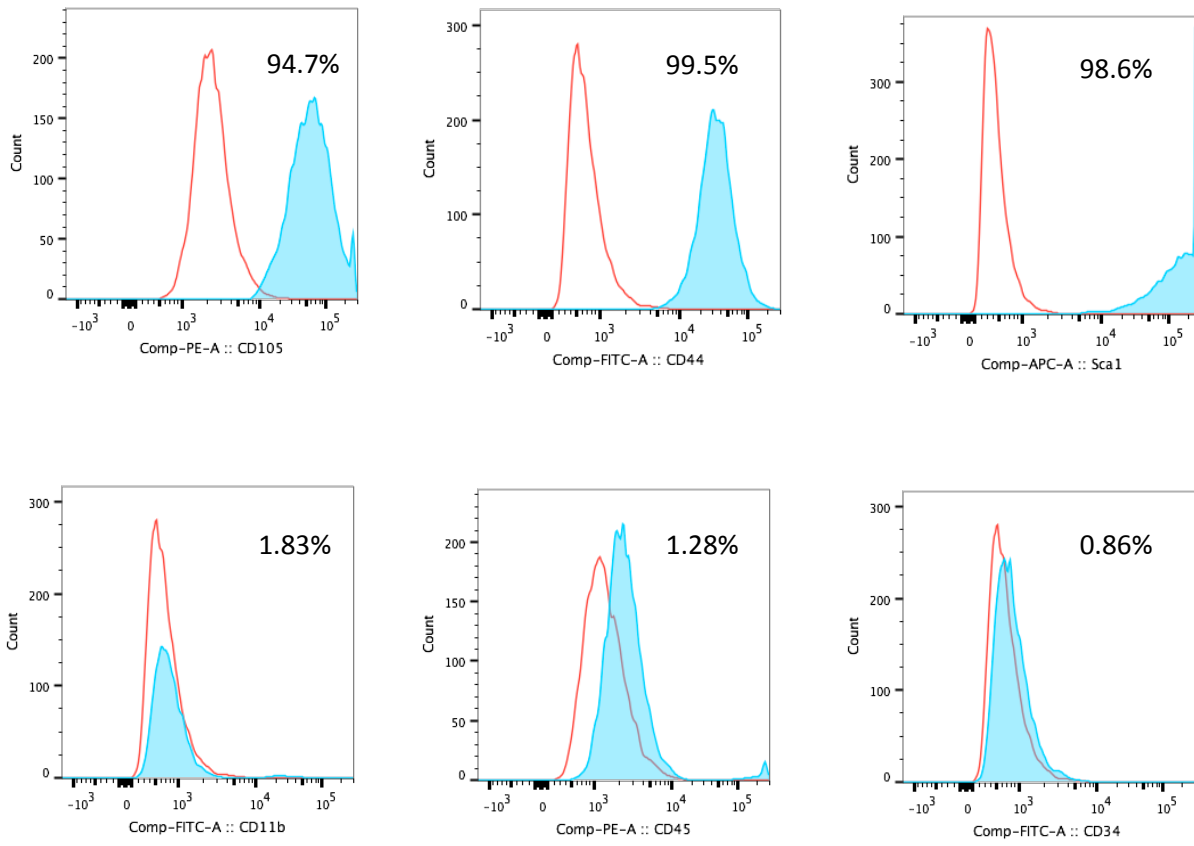

Figure S2

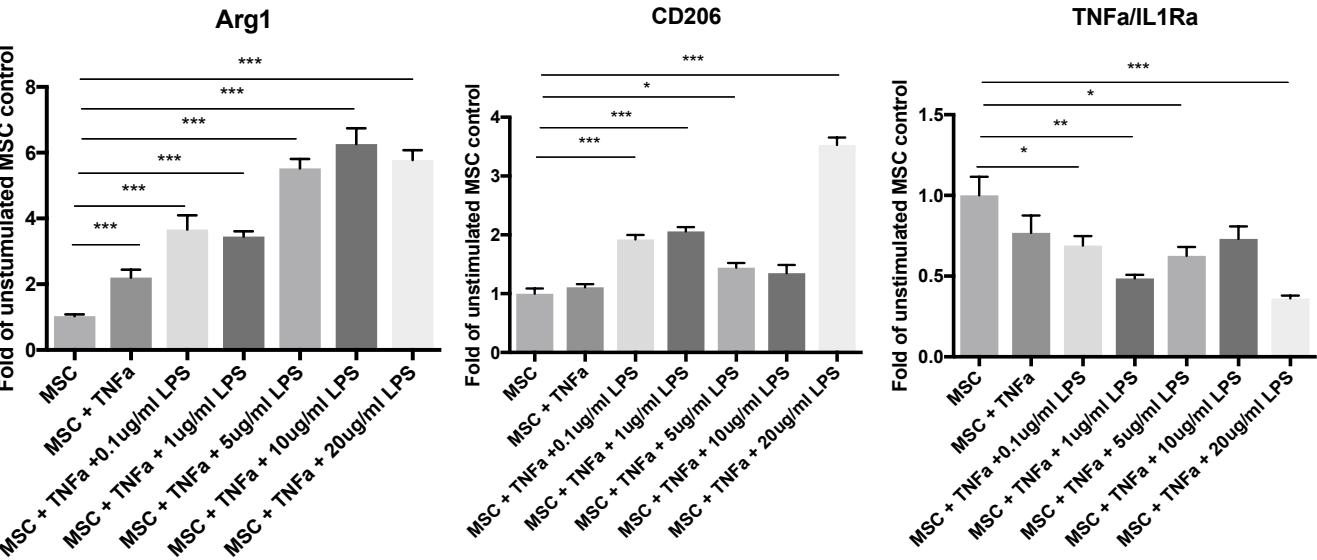

**Figure S3**

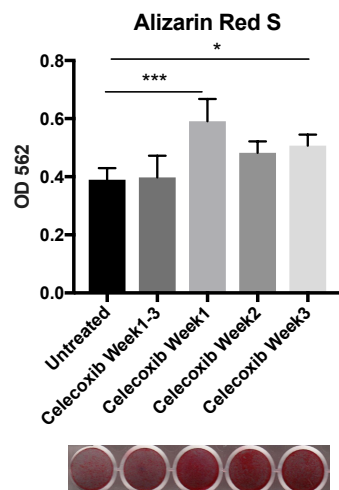

**Figure S4**

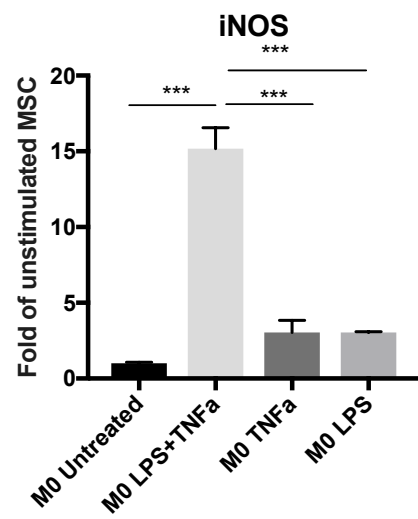

Supplement: Additional file 1: Figure S1. — Showing immunophenotypes of murine bone marrow-derived MSCs. Surface marker expression in murine MSCs at passage 4 analyzed by flow cytometry. Figure S2. showing titration of LPS dose for MSC preconditioning with TNF-α to modulate murine macrophage polarization. Murine MSCs were preconditioned with TNF-α (20 ng/ml) plus LPS (1–20 μg/ml) for 3 days, and cocultured with M1 macrophages for 24 hours. M2 (Arg1 and CD206) and M1 (TNF-α/IL-1Ra) macrophage marker expression in macrophages measured by quantitative PCR. Data presented as fold-change compared to macrophages cocultured with unstimulated control MSCs. *p < 0.05, **p < 0.01, ***p < 0.005. Figure S3. showing increased osteogenic differentiation in the MSCs with temporal inhibition of COX2 signaling. MSCs were treated with 25 μM Celecoxib in osteogenic media at indicated time points. Osteogenic differentiation ability examined by bone mineralization (Alizarin Red staining) at week 3. *p < 0.05, ***p < 0.005. Figure S4. showing increased iNOS expression in MSCs preconditioned with TNF-α and/or LPS. Murine MSCs were preconditioned with TNF-α (20 ng/ml) plus LPS (1–20 μg/ml) for 3 days, and washed out for 24 hours. Expression of iNOS determined by quantitative PCR. Data presented as fold-change compared to macrophages cocultured with unstimulated control MSCs. ***p < 0.005. (PDF 3973 kb) [file 13287_2017_730_MOESM1_ESM.pdf]
